# Supplementary material for: Genetic characterization of a new radish introgression line carrying the restorer gene for Ogura CMS in Brassica napus
Source: PLoS One. 2020 Jul 28;15(7):e0236273. doi: 10.1371/journal.pone.0236273 (PMC7386589; doi:10.1371/journal.pone.0236273)
Supplement: S1 Table — (DOCX) [file pone.0236273.s001.docx]

**Additional Table 1**. Sequence of amplified PCR products from self-designed SSR markers from Table 4.

| Marker Name | Sequence of amplified PCR products（5’→3’） |
| --- | --- |
| CLR9-1 | GGCTCAACGCGGAAAGCAGATGCTCTGCAAGGACACTCACCTGCGGTCTTTCCTATTGAGTCTCTCTACCTTTAATTGAATAGACTTCCATTTCTTTGGACAGATTGTAGCCTGTCCTGTGTGTGGTTGACTCCTCGTTTTCTTTGATTATTATTGTTATCAATCACACGCAGAGTGACAATCGGCACAGAGAAATATTTATTTCGTTTGTCGGCATGTGACCGTCCCCAAAGAAAGAGAGTGATCGAGATTCAATTCGACATGGAGTGTCTGCTGTGACTGTGCCCTGGACATCGGAGTGCCTCAATTTCAACGAATACACA |
| CLR9-2 | AAGTTCGGACGAAAGAGAGGGCAAGTTACCTCTTGAAGGTTTGACCTCCATCCTTTCACCGCTATCAAGGAAGAAAATGACCATATATTTGTTTGGGCACCTGATTTTACACCAAGACATTTTCACTAAAAAAAAAAAAAAAAGTGTCCCTTTTCGGGGTTATTGGGGGTTTTTATTTTTCCCCGTAAGGTTTTCCGGGGATCCCCTTTAAAAAATAAAAAAAAGTGATTGCGTCCGGTTTGAAATACACCTGGCCACCCAGACGTAACCTACGACGGGTTTCAATCTTTTTCAATGTTTTAAAGCAATCATATTAATACCGTAACTAGGTGAGACCGGTGGGTAGGAGGGATT |
| CLR9-3 | GCCGCGAGTAGAGGAAGAAGAGCTTATCTAAAAACTAACTTGCCTTGTCTAAGCTCCTGATTAACCGGTATAATTGTTTCTGGAGAATTTAAAAATACTGATCTAAATGCTTAAATATTTGGAATACTCTCTTTAAAAATTTCCAAAATATTTTTTAAATTATTATTGAAATTTTTTAACGTATAATATCCCGCGCTTCTTAAGCTCGGATCAAAATCTTTTAATTTTATCCATTAACACATAACCCTGGCCACCCCGACAAAAGCTACGACTGGTTTCCGTTTTTTACTATGTATTAATGCAATCTTATTATTATTATTAACTAGGTAAGACCGTTGCGATATGGAATTCCAATCCCCCTAGAGAATAAAACTCCTCCAATCCAAACCCAAGGTGGGGGTCAACCGAATTCGGTAAACCCCGGCCGGTAAACAAAGAA |
| CLR9-4 | CAGGGGGGACAACAAACAAATATGAGTAATTACCAAGATCTTTTTTTTTTTGCCCCCCATTGCCCCTGAAATTTGTTCAATTTCCCCCCTGTCTAACTATTTGGGAACCCCCCCTTATGCCCGAACCCCAAAAAACCGAAAGGCCCCGCGGGGGGGCTCCCCCAAGGGGGGACCCCCCCCCCGCGCCTCCCCGTTTTTTGGGGATCCCCCCCTACATGGGGCCCCCCCCCAACTTAACCCACGGCCGGATTTTAACCCTTTTATTTTCTTCCGGGGCCCAAAAAAAAAAAAATTTGGGAAAAAAAAAAATTTTTTTAAATTTTTTGGGGGGGGAAACTTTTGTTTTCCCCAAAGTGGAAAAAAA |
| CLR9-5 | TCGGAAAGGGCAAGAAGCAAGTTGCTGTTTAATTCCGGATCCTTTTTGCTTCGTCATTTTATATTGATTTACAAAATTTCTTTAAATGTCTACTTTAATTGGGCTCCCTGAGGTTTTCCCAGTTAGGGCCCTTACTGGCAAACACATCCAGAGCCCAATTCCAGTTTACCCTTAATTTAACTTTTTCAAATCAATTTAAAAAGAAGATTATCAAAGGGGTCCGGCGTGAAATCAGCCCCGGAAAAAAAAAAACAAAAGAAAAAAAAATCTGGAACAGCGCTGCCCCACGAGGATTATCGGGGTGCCATGACTTCACATAAAAACATGAACTAGGTAATACCCCGGCCAAAATGGAAAAAAAAATGAT |
| CLR9-6 | TCGCGAGGTGCAAGAGGGAGGCAAGGACGCGTAAAAAGTACTGGTATGGTATTAATTCGCGTAGAATAAGTGACAAAATATTTAGTTTTAATATGGCACACAAGATTAGACAAGAAGACGTTGACGAATAAAAAAAAAAAAAAAAAAACTTTAACGGGGTCGTTGGGGGTTTAAATTTTGGTTTTTACCCTTTCCCCGGAAAACCCTTTTTAAAAAAAAAAAAAAGATCCATCATGTAGGACTTTGACCTGGCCACCCAGACAATGCAACGACTGGTTTCCTTTTTTACTATGTATTAATGCAATCATATTATTACCATTAACTAAGTAAGACCCTGCCAAAATGGAAT |
| CLR9-7 | CCGAAGGGAACAAAAGAAGGAAAACGAAAAATAAAAAAAAGAAGTTAAGACATCTTTTTCTTTTGTTAATATAAATAAACAACCAGACCAACATTGATCTGCCTTTTCCTTTGTGGATTAAAATCAATTGGGCCACCAAAATCAAATAATCCCCAATCATGGGTATTAATCCCTTCTGGAGGCCCCACTCAATTTTGCCCAACAAAAAAATCGCTTTACTTTTTTTGCTTTGTTTATTTTTTTATATTACCAAAAAAAAAAAATTTTTTCCCCATTTTAATTAAAAGGGAAAAGCGGGAAAATAAAAATTTCTTTTTTTTTTATTATGGGGTCTAACCCTTTTCCTTTGGTTTAAATTAAAAAA |
| CLR9-8 | CCGATCGGAAAAAGCTGAAGGACGTTCAATCCTTATTCCCTTTTTGATTTGTAAACTATGGCATCTATTTTTTTTATTAATATTAACAAACAAACAAAAAAAAATTGATCTTGCTTTTCCATTGATGATCAAAATCGATTGGACGACCAAGACCAGATTAATACCCGACATCTGCGATTATCCATGAAGGAGGCACAACTCAAATTATCACAACAAGAAAAATGCTTTACTTTTTGTGCTCTGCTTATTTATTTATATAACCAAAAAAAAAAAATTTTTTTCCCTATTTAATTAAAATGGAAAAAAAGGAAAAACAAAAATTCCTTGTATTTTATTATTGTGTCTTACCTTTTTTCCTTGTTTTCCAATAAAAA |
| CLR9-9 | GCGCCGCGCGAAGAGAGAAAGCGCAGTGTATGTTAGTGATGATATAAACTTCTATTTGAGGCTGATGCCTGAAAAAAATTGGATATTTTCTTATACTTTCGATATTTTGGATAAAACATATTAGGAAAATTTTGGATGATCAAATATTTGATTCTACTTTAATTTTTCAAATATGTTATATCTTTTAAATACATTTTTTTTTTTTATATCTATTTTTTGTTATGCCACTACCTCATATGTATGTTTATATCTTCTCTTTCTACATTCCCATTGAGGGGATGTGCGAGGCGGGGATTGTGTAAGACGTACAACTCTCCTGTTTCGCTCTTTGATTTTGGGCGGGGGGGTCAACCCGTTTTTCTGGGGATCAAAAAAAAAAAAAGGGGGGGGGG |
| CLR9-10 | TAGGAGACGGGAGAACCAAACTGTGAGTAATACTGCGTGAGAGAAATGGTCTTCCTGCTCAGATCTCTCCCTTTCACTAAAGATAAATAAACCCTAAAACTTTCCCGCAGTTTCTTTCTCCTTCCGAGTTTCCCTAAAAGTACGAACTACTCCTCTTCAAAGACCCATCTGCTTGAATTACCAAAATCATCAGCAAAAATACCCAGGTAAAAATTACTGCTTTTACTTCTTCATCTTTATAAGGGCCTTTCACTTTTTTTTTTTTTTTTTCCCGTTTTTGGTTTTTTACCCGGGGGGGGGGGGTTTTCCCTTTTTAACTCTTTCCCTTAAAAGGGGGGGTCGAAAAAA |
| CLR9-11 | TCCGAGCCGAGAGAAAGAAGAGTGCAACGAATTTCATCGAACTGGTCGTCCTCCTTTGGGCGGCATGACTCCTCCTCTTTCTTCATCCATCTTGTCTTACGTCGCTTCGACTCTCGCGGGGGGTTCTTCTTCCTGAACCTCTCACCCATTCATCTCTCCTTCCTTTTTTCCCTTACTCTCTCTCTCTCTCTCTCTATCTCTTCCTCCCCCATAGACGTGCTTCACGTTGTTAGGTTCTTTCATTTTTTCCTGGAACATAAAGAATACACGTGTCTTCTTCCTCACCCCTCCCGTTATTTATAAAAAAAGATTTCTTTGTATTTTATAGGCGGGGGGGCCCCCTTTCCTTTTCGAGTGCAAAAAAAAAAA |
| CLR9-12 | TCGGTGCTGGTAGGCGGGAACTTTACCTCGATCCTAAGGGTATATTTCAAGATTGCTGTAAGTTCGGTGACATATATATGATAATATATTGTTCCAATTTTTTAATATATATATATATATATATATATATAGAGAGTGTATTTAAATTATTTTTTTACTGTATACCCATCTGAAAAAAATGTGTACTTGATTTTCGTACAAAAATTACTCTCCCTCCCGTTTCTCCGGGGGGGTATATAAAAAAATCTATCCTGGGATGGCCCCCGCCCTCTAAATATAGGGTTTAAAGGAAATTTCAAAACCGTTACGTTTTTTACGCCCCCCGCCCCCCCCGGTTTCCGGGGAGTAAAATAAAAAAAG |
| CLR9-13 | TACAATATAAATGAAATTTTATTTGTGATCTTGCCGCTCCCAACTCGAGCGAAACCAGGTACGCAAAACGTTACATATATGTTAGGTTGTTGCCCTTTGGCCCAATTTTTTCTAATTTGCCCCTCCCTTTAAAAAAAAACCAAATTAAAACCGCCTTTAAAAAAAATTTTCCCCCAAGACGGGCAAAAAAAAAAAGGGGTTTTTAATTTTTTTTCAAACAAAAAAAATTTGCCAAAATTTTTCTTTGTTTCTTTCCAAAAAAAAACCATAATAATTTTGAATTTTTTTTGACCCGGTTTCCCCCCGAGGGGGGGCCCCACAACCCGCCCCCCCAGGGGGGGGAAAATTCCCCCCCCCCTTTTTGAACCGACATAAGGGAAAATTT |
| CLR9-14 | TAACTATAAAAAATTTTTTCCGCGTTACCACTCTTATACTACCAGTTGCTTATGGTAAACTACTATTGGATAGTAATCTAGTGGATTTATGCTATTCTGCCTACTGGTGGTTTTCCTTGGCTGTTGAAAAAAAACCCCGAGGGGACGAAGAAAAAACATGTATCTATATATATATATATATATATATATATATACATATATCTATATACCCATATATCCAAAACCCGGGTTGTCTTTTTTCTTTCTCACAATACATTATTACAACAGGGACACTTTTTCCTCTCGCTCCCAATCAAACGGGGGAGAAGGCACCAACAAAAAAAAAAAAAAAGGAGCCTGGTCTCAAGCCGATTAAAAAGGGGCCAAT |
| CLR9-15 | GAAGGTTAAAGATAAGACGAGCTTGCTTTGCAGGAGAATCTATGGAATTCCTTAAGCTTGGCCGACGAACCCGAAGCCTGAGGACACGCCGCTTTCTCCTCCGGCGACTTCGCCTCCGCCGTCAACCACTTCACCGAAGCGATCAACCTCGCCCCGACCAACCACGTCCTCTACTCCAACCGCTCCGCCGCCCACGCCTCCCTCCTCCGCTACGAGGAAGCTCTCTCCGACGCCAAGAAGACCGTCGAGCTCAAACCCGACTGGGCCAAGGGCTACAGCCGCCTCGGCGCCGCTCACCTCGGCCTCAACCAATCCGACGAAGCCGTCGAGGCCTGCTCCAAGGGTCTCGAGATCGATCCAAGCAACGACGCGCTTTAATCGGGTTCTGCAGACGGCTTCGAGGGTCCGCGCCGCCCCTCCCCCCCCGAATCCGTTTGGGGACGCGTTCAAGGGGCCAGAGCATGTGGGCGAAGGTGAGGGCGGATCCGTCGACGAGGGGGTTCTTGTGTGAGCCTGACTTCGTCAACATGATGCAGGAGATGCAGAGGAATCCTATAACGGGAAAA |
| CLR9-16 | TAAGGCATAGGCAGAAGAGCAACTGCTCAAGATTTCTACTTAAACTGAAACATTTTCTGTGACACACTCATTGTAGCCGGAAGAAAAGAGATTGTCGTATCTATTCACTCCTCACAGCAGCTCTCTCGATCATGAACATGACCACAGTGAGAGAGAGAGAGAGAGAGAGATATCCAAATTTACTTTCACTATCTCTCCTTCAAGTTTTTCAGATGCTTCATTTGCTTCCTCTTGACTTTAAAAACAACTTCGGCGCTATATCAACCTCCTACCTTCTTTGGTGGCTGCACAAACATGATCCTCTCTACGAA |
| CLR9-17 | TAAATATTTTTCTTTTCTCCGGATAGATACTCCAGGGAGTCGTGCAAGTGGCCGACTCTGTGCGCCCACTCGGGCAGAGAAGGCGGGCCTCAATTGGTGGTTTTCGTGGCTGTGCTTGTATGATTGTTGACTTTCGGTTGTCTCCATGTCTCTTTGTGATATGTTCTTCCTCCAGGAATTTCCCCCAGCTATTGACCAAGATATCCCCGGCCTGGAGGTTCTTTTTTCTTTTTCACGATACGGTACTACAACAGGCAGATAGGATTCCAGTGTCTCCCTCACAACGAAGAAGCTTATTCCGTTGCTGAGTTTGCTCAGGAGCCTGGTCACAAGCGGGATGAGAAGG |
| CLR9-18 | TACGGAAACAAGGGACGAAGAGCAATGGAGTCAACAGATGACTTATCTGTTGCTTTTTTTTGAAATCCTCACAAAAATCCGAAGAACAACCGAACTGAAAACATATGAGTTTTGTAGTTTCCATAATTGATTTCATCAATCGAACCTAAAATATAATTTTTTCTTGGTCTGCTGATGAAATCCAATATTAGAATCACAAAATCTCTATATCTCTCATTTCGTTTTTCTTTTTATTTATGATAAATTATTATTTTTAAAAATTATTGATAACTTTTTCATGAATAACTTGTTTTTTTTTTGGAACGTCTTTGAGAAAAATAATGTGCCATATGTTGTACCGAAAAAA |
| CLR9-19 | TCCGGGAAGGAAAGCGAACATGAACCGTTGTAAACGATATCTTATCTGTTGCTTTTTTTTGAAATCTTCACAATAATCCGAACAACAACCGAACAGAAAACATATGAGTATCGTAATATCCATAATTGATTTCATCAACCGAACCTAAAATATAATTTTATCTTGGTCTGCTGATGAAATCCAATATTGGAATAACAAAATATATATATCTTTTATTTCGTTGTTCTTTTTATTTATGATAAATTATTATTTTTAAAAATTATTGATAACTTTTTCATGAATAACTTGTTTTTTTTTTGGAACGTCTTTGAGAAAAATAATGTGCCATAGGTTGTACCGAGGA |
| CLR9-20 | TACACAAGAAAAGCGAGACAGAGAGTTCTTTTAGTATTTTGACGGGTACTTAGACTTTCATACTTCTATTACCTAACGTTTTGGAAGAATAACAATCTCTACTCCATGTGAGGAGCTAGTATTAAGTGTAAGGAAAAATTTCTTCCGAAAGCGCTCTGTCTCTCACTCTATATAGATAAATATATAGATATTTAAATATTTATATATAAATATCCAGATTTGTAAAAAATTAAAAACCTTCCCATTTTCAATTTCGGAAAATCAAAAAAATTTTTTTTTTTTTATATATATATATATATATATAAAAAAATAAAAAAATATATATATATATATATATAGAGAGAGACGTGTTCCACTCTCTCAAAAAAATATCTATATATATATAGAGAGACAAAAGAGAGAGAGAGAGTGTGTGTCACTCCCACACACACTCGAAAAAGAGAGAGAGTGTGTGTGTGTCTCTGTGACAAAAAAAAATTTTGTGAGACACTGTGTGTATTTTTATCAACACCCCCTCTCTGTGTGTATAGAGAAA |
