# Supplementary material for: Genetic characterization of a new radish introgression line carrying the restorer gene for Ogura CMS in Brassica napus
Source: PLoS One. 2020 Jul 28;15(7):e0236273. doi: 10.1371/journal.pone.0236273 (PMC7386589; doi:10.1371/journal.pone.0236273)
Supplement: Attachment — Submitted filename: PONE-D-20-03673_reviewer.pdf [file pone.0236273.s002.pdf]

PLOS ONE

Genetic characterization of a new radish introgression line carrying the restorer gene for Ogura CMS in Brassica napus

--Manuscript Draft--

**Manuscript Number:**	PONE-D-20-03673
**Article Type:**	Research Article
**Full Title:**	Genetic characterization of a new radish introgression line carrying the restorer gene for Ogura CMS in Brassica napus
**Short Title:**	Characterisation of a new Ogura CMS restorer line
**Corresponding Author:**	Yiming Guo<br>Hunan Academy of Agricultural Sciences<br>Changsha, CHINA
**Keywords:**	
**Abstract:**	p.p1 {margin: 0.0px 0.0px 0.0px 0.0px; line-height: 19.0px; font: 13.0px 'Helvetica Neue'; color: #000000}<br>Creating a homologous restorer line for Ogura cytoplasmic male sterility (Ogu-CMS) in Brassica napus is meaningful for the wider application of Ogu-CMS system in rapeseed production. Previously, an independent development of a new Ogu-CMS restorer line (CLR650) was reported locally from crossing between Raphanobrassica (AACCRR, 2n=56) and B. napus and a new version of Ogu CMS lines CLR6430 derived from CLR650 was characterized in this study. The results showed that the fertility restoration gene in CLR6430 presented a distorted segregation in segregating population. However, the majority of somatic cells from roots had a regular chromosome number (2n=38) and no radish signal covered a whole chromosome was detected using GISH. Thirty-two specific markers derived from the introgresssed radish fragments were developed based on the re-sequencing results. Unique radish insertion and differences between CLR6430 and R2000 were also identified through both radish-derived markers and PCR product sequences. Further investigations on the genetic behaviors, interactions between the fertility restoration and other traits and specific molecular markers to the introgression in CLR6430 were also conducted in this study. These results should provide the evidence of nucleotide differences between CLR6430 and R2000, and the specific markers will be helpful for breeding new Ogura restore lines in future.
**Order of Authors:**	Yiming Guo
	Tonghua Wang
	Zengxiang Wu
	Shengqian Xia
	Shuijin Hua
	Jinxin Tu
	Mei Li
	Weijiang Chen
**Additional Information:**	
**Question**	**Response**
**Financial Disclosure**<br><br>Enter a financial disclosure statement that describes the sources of funding for the work included in this submission. Review the submission guidelines for detailed	Funding for operating costs of this research was provided both by the National Natural Science Foundation for Young Scientists of China (Grant No. 31401467) and The Key Research and Development Program of Hunan Provinces (Grant No. 2016NK2200).

Powered by Editorial Manager® and ProduXion Manager® from Aries Systems Corporation

requirements. View published research articles from *PLOS ONE* for specific examples.<br><br>This statement is required for submission and **will appear in the published article** if the submission is accepted. Please make sure it is accurate.<br><br>**Unfunded studies**<br>Enter: *The author(s) received no specific funding for this work.*<br><br>**Funded studies**<br>Enter a statement with the following details:<br>• Initials of the authors who received each award<br>• Grant numbers awarded to each author<br>• The full name of each funder<br>• URL of each funder website<br>• Did the sponsors or funders play any role in the study design, data collection and analysis, decision to publish, or preparation of the manuscript?<br>• **NO** - Include this sentence at the end of your statement: *The funders had no role in study design, data collection and analysis, decision to publish, or preparation of the manuscript.*<br>• **YES** - Specify the role(s) played.<br><br>* typeset	
**Competing Interests**<br><br>Use the instructions below to enter a competing interest statement for this submission. On behalf of all authors, disclose any competing interests that could be perceived to bias this work—acknowledging all financial support and any other relevant financial or non-financial competing interests.<br><br>This statement **will appear in the published article** if the submission is accepted. Please make sure it is accurate. View published research articles from *PLOS ONE* for specific examples.	The authors have declared that no competing interests exist.

Powered by Editorial Manager® and ProduXion Manager® from Aries Systems Corporation

**NO authors have competing interests**<br><br>Enter: *The authors have declared that no competing interests exist.*<br><br>**Authors with competing interests**<br><br>Enter competing interest details beginning with this statement:<br><br>*I have read the journal's policy and the authors of this manuscript have the following competing interests: [insert competing interests here]*<br><br>* typeset	
**Ethics Statement**<br><br>Enter an ethics statement for this submission. This statement is required if the study involved:<br><br>• Human participants<br>• Human specimens or tissue<br>• Vertebrate animals or cephalopods<br>• Vertebrate embryos or tissues<br>• Field research<br><br>Write "N/A" if the submission does not require an ethics statement.<br><br>General guidance is provided below. Consult the submission guidelines for detailed instructions. **Make sure that all information entered here is included in the Methods section of the manuscript.**	N/A

Powered by Editorial Manager® and ProduXion Manager® from Aries Systems Corporation

**Format for specific study types**<br><br>**Human Subject Research (involving human participants and/or tissue)**<br>• Give the name of the institutional review board or ethics committee that approved the study<br>• Include the approval number and/or a statement indicating approval of this research<br>• Indicate the form of consent obtained (written/oral) or the reason that consent was not obtained (e.g. the data were analyzed anonymously)<br><br>**Animal Research (involving vertebrate animals, embryos or tissues)**<br>• Provide the name of the Institutional Animal Care and Use Committee (IACUC) or other relevant ethics board that reviewed the study protocol, and indicate whether they approved this research or granted a formal waiver of ethical approval<br>• Include an approval number if one was obtained<br>• If the study involved *non-human primates*, add *additional details* about animal welfare and steps taken to ameliorate suffering<br>• If anesthesia, euthanasia, or any kind of animal sacrifice is part of the study, include briefly which substances and/or methods were applied<br><br>**Field Research**<br><br>Include the following details if this study involves the collection of plant, animal, or other materials from a natural setting:<br>• Field permit number<br>• Name of the institution or relevant body that granted permission	
**Data Availability**<br><br>Authors are required to make all data underlying the findings described fully available, without restriction, and from the time of publication. PLOS allows rare exceptions to address legal and ethical concerns. See the PLOS Data Policy and FAQ for detailed information.	Yes - all data are fully available without restriction

A Data Availability Statement describing where the data can be found is required at submission. Your answers to this question constitute the Data Availability Statement and **will be published in the article**, if accepted.<br><br>**Important:** Stating 'data available on request from the author' is not sufficient. If your data are only available upon request, select 'No' for the first question and explain your exceptional situation in the text box.<br><br>Do the authors confirm that all data underlying the findings described in their manuscript are fully available without restriction?	
**Describe where the data may be found in full sentences. If you are copying our sample text, replace any instances of XXX with the appropriate details.**<br><br>• If the data are **held or will be held in a public repository**, include URLs, accession numbers or DOIs. If this information will only be available after acceptance, indicate this by ticking the box below. For example: *All XXX files are available from the XXX database (accession number(s) XXX, XXX.).*<br>• If the data are all contained **within the manuscript and/or Supporting Information files**, enter the following: *All relevant data are within the manuscript and its Supporting Information files.*<br>• If neither of these applies but you are able to provide **details of access elsewhere**, with or without limitations, please do so. For example:<br><br>*Data cannot be shared publicly because of [XXX]. Data are available from the XXX Institutional Data Access / Ethics Committee (contact via XXX) for researchers who meet the criteria for access to confidential data.*<br><br>*The data underlying the results presented in the study are available from (include the name of the third party*	The data discussed in this manuscript have been deposited to NCBI's SRA database with BioProject, number PRJNA488570 and is accessible through the website linkage at http://www.ncbi.nlm.nih.gov/sra/SRP159151.

Powered by Editorial Manager® and ProduXion Manager® from Aries Systems Corporation

and contact information or URL).<br>• This text is appropriate if the data are owned by a third party and authors do not have permission to share the data.<br><br>* typeset	
Additional data availability information:	

Main file Click here to download Manuscript 2020_Ogu paper_plos one.docx

Genetic characterization of a new radish introgression line carrying the restorer gene for Ogura CMS in *Brassica napus*

Tonghua Wang[1,+], Yiming Guo[1,+], Zengxiang Wu[2], Shengqian Xia[2], Shuijin Hua[3], Jinxing Tu[2,*], Mei Li[1,*], Weijiang Chen[1]

[1] Crop Research Institute, Hunan Academy of Agricultural Sciences, Changsha, China;

[2] National Key Laboratory of Crop Genetic Improvement, Huazhong Agricultural University, Wuhan, China;

[3] Institute of crop and nuclear technology utilization, Zhejiang Academy of Agricultural Sciences, Hangzhou, China;

* Corresponding author

Tel: +86 13787111618;

Fax: +86 731 84691287;

Email: limei1230@126.com; tujx@mail.hzau.edu.cn

[+] These two authors contributed equally to this work;

Abstract

Creating a homologous restorer line for Ogura cytoplasmic male sterility (Ogu-CMS) in *Brassica napus* is meaningful for the wider application of Ogu-CMS system in rapeseed production. Previously, an independent development of a new Ogu-CMS restorer line (CLR650) was reported locally from crossing between *Raphanobrassica* (AACCRR, 2n=56) and *B. napus* and a new version of Ogu CMS lines CLR6430 derived from CLR650 was characterized in this study. The results showed that the fertility restoration gene in CLR6430 presented a distorted segregation in segregating population. However, the majority of somatic cells from roots had a regular chromosome number (2n=38) and no radish signal covered a whole chromosome was detected using GISH. Thirty-two specific markers derived from the introgresssed radish fragments were developed based on the re-sequencing results. Unique radish insertion and differences between CLR6430 and R2000 were also identified through both radish-derived markers and PCR product sequences. Further investigations on the genetic behaviors, interactions between the fertility restoration and other traits and specific molecular markers to the introgression in CLR6430 were also conducted in this study. These results should provide the evidence of nucleotide differences between CLR6430 and R2000, and the specific markers will be helpful for breeding new Ogura restore lines in future.

Key words: cytoplasmic male sterility; resequencing; genomic introgression; marker-assisted breeding

Introduction

Cytoplasmic male sterility (CMS) is a widespread inherited trait in plants, which is controlled by an incompatibility between the nucleus and mitochondrial genes and has been widely used for F_1 seed production. Some CMS systems have been identified in *B. napus*, such as Polima CMS (*pol* CMS) (1), Ogura CMS (*ogu* CMS) (2), the common *B. napus* CMS (*nap* CMS) (3), and a novel type introgressed from Chinese woad (*inap* CMS) (4). Ogu CMS was first discovered in Japanese radish (*Raphanus sativus*) from an unknown cultivar (Ogura 1968), and the sterility of o*gu* CMS was stable in various climate conditions (5), which has been utilized more and more widely.

The application of Ogu CMS has been difficult as the restorer gene (*Rf*) only existed in some Europe and Japanese wild radish, but not in *B. napus* nor any *B. rapa* varieties. Therefore, the *Rf* gene had to be introduced from radish into oilseed rape through intergeneric hybridization. Homologous recombination between R and A and C genome were achieved through polyploidy cross (6), gamma irradiation of donor pollen (7), or asymmetric protoplast fusion (8). However, along with the introduction of the *Rfo* gene into *B. napus*, extra segments of radish genome was introgressed into *B. napus* which brought undesirable traits for breeders, such as elevated glucosinolate level and poor seed set (the number of seed per silique) (9). The early version of *Brassica* Ogu restorer lines developed by INRA identified a close linkage between glucosinolate content and the *Rfo* gene (10), suggesting more difficulties in breeding double low varieties. Therefore, following research was dedicated for the improvement of the restorer line. Delourme (11) reported a low glucosinolate (9-18 µmol/g) Ogu-CMS restorer line in *B. napus* assisted by RAPD markers, however the *Rfo* loci was

heterozygous and not heritable to siblings. Successful break-through of low glucosinolate Ogu-CMS restorer line was obtained through gamma radiation when crossing with *B. napus* of low glucosinolate content, and named as R2000 (7, 11). Other independent breeding of *B. napus* Ogu restorer line using radiation or other methods were reported by Pioneer Hi-bred Company and Syngenta Biotechnology Company (12-14), with shortened radish fragment in new generations.

The *Rfo* gene, *orf687*, encoding a 687-amino-acid protein with multiple pentatricopeptide repeats, alters the expression of the sterile gene *orf138* at post-transcriptional level (15, 16). The gene has been localized physically and mapped in the distal region on linkage group N19 in Pioneer-derived Ogu-CMS restorer line, which corresponds to the C genome in *B. napus* (17). Moreover, apart from the identified *Rfo* gene (15), other alleles were identified by Wang, Zhang (18) and Wang, Zhang (19), and was 1.6 cM distant from *Rfo* locus in Ogu-CMS radish. Further investigations of these alleles revealed a heterozygous type (RsRf3-1/RsRf3-2) encoding PPR proteins were responsible for the fertility of male-sterile radish, and had higher expression and RNA polymerase II occupancy compared with their homozygous alleles (RsRf3-1/RsRf3-1 or RsRf3-2/RsRf3-2) (20). However, those new identified alleles were studied only in radish. Therefore, associations between these alleles and the *Rfo* gene and their independent functions when transferring into *B. napus* remain unclear.

The introgression of the *Rfo* gene from radish into rapeseed also introduced chromosomes or large amount of radish genome fragments around the *Rfo* locus and possibly elsewhere in the genome. The redundant introgressions are more likely to lead to deleterious genetic characteristics such as poor agronomic performance, increased glucosinolate content, and

distorted segregation. The type of cross and mapping population can influence the incidence of genomic regions exhibiting distorted segregation, not to mention the exogenous introgressed or inserted material. However, not many studies linked this aspect to the radish-derived Ogu CMS in *B. napus*, the genetic behaviors of Ogu restorer line as pollen donor or accepter remains unclear and is worthy of investigation. Moreover, molecular marker assisted breeding has been applied throughout the breeding history of Ogu-CMS restorer line in *Brassica* family (11, 21-23). *Rfo* specific markers in canola were developed by Hu et al (21), while Tian et al (22) and Yu et al. (23) applied to *B. juncea* and *B. oleracea* respectively, enabling previse selection for low glucosinolate within larger population.

Previously we reported a newly-developed Ogu-CMS restorer line (CLR650) from crossing between *Raphanobrassica* (AACCRR, 2n=56) and *B. napus* using grafting to overcome the incompatible obstacle between these two parents (24). Further analysis of the updated version of material (CLR6430) developed using the restorer CLR650 through backcross with double-low line and self-pollination was conducted in this study to investigate the chromosomal behaviors and fertility ability. Resequencing was also applied to CLR6430 to explore the detailed information during the introgression from radish fragment into *B. napus*, enabling more precise designs for marker-assisted breeding for the new Ogu-CMS restorer lines application.

Materials and Methods

Plant material

Using *Raphanobrassica* (AACCRR, 2n=56) as the donor of Ogu CMS restorer resource, we

successfully obtained the hybrids of *Brassica* with *Raphanobrassica* through grafting to overcome the incompatible obstacle between these two parents. After 15 years with high intensive selection, a stable homozygous restorer line CLR650 of Ogu CMS in *B. napus* was bred which can restore the male fertility of progenies from self-pollination and testcross (24). A new version of Ogu CMS restore lines CLR6430 derived from CLR650 through 2 generations of backcross with double low line 20B and then 4 generations of self-pollination.

Segregating populations preparation and fertility identification

Segregating populations were generated using two Ogu CMS sterility lines (A-line) 20QA and SC3QA (*rfrf*) and were crossed as female with CLR6430 respectively and selfing for F_2 and F_3. These two lines were also utilized for back-cross (BC_{1-2}) populations and selected for low glucosinolate content concurrently. Reciprocal crosses were conducted between CLR6430 and a general selfing line 20B (The maintainer line of 20QA) and SC_3 (The maintainer line of SC_3QA).

The fertility identification of all segregating populations was performed around 11 AM on sunny days during flowering stage of plants three times using 1% acetocarmine under a microscope (DS-Ril, Nikon, Japan). And a F_2 population of 192 individuals derived from the cross of 20QA×CLR6430 was used for DNA marker testing.

Chromosome detection

Newly-germinated seed roots and ovaries from young flower buds were used to determine the chromosome numbers of CLR6430. The first 2-3 mm root tips and ovaries were treated with 2 mM 8-hydroxyquinoline for 3-4 h at room temperature and fixed in ethanol: acetic acid (3:1, v/v) before stored at -20℃. Well-separated chromosome slides were used for chromosome

counting (DS-Ril, Nikon, Japan) and then stored for further genomic and fluorescence in situ hybridization (GISH) and fluorescence in situ hybridization of bacterial artificial chromosome (BAC-FISH).

GISH and BAC-FISH analysis

Plant DNA was extracted from the newly-grown young leaves using DNeasy Plant mini Kit (Qiagen, Hilden, Germany). The BAC clone of restorer gene used *CopyControl*™ *pCC1BAC*™ *(Hind III Cloning-Ready) Vector,* and the concentration of chloramphenicol resistance was 12.5μg/mL, with average 95kb inserted in the constructed library. The C genome of *Brassica napus* and BAC clone was labeled by random priming with biotin-11-dUTP (Sabc, China) and digoxigenin-11-dUTP (Roche, Basel, Switzerland). The A genome of *B. napus* was sheared by boiling for 15 min and used as a block.

The photographs of well-separated chromosome slides were taken under the fluorescence microscopy following the methods describing by Ge and Li (25).

Collection of re-sequencing sample, DNA extraction and sequencing

Seeds from CLR6430 line (46608, double confirmed with restorer gene) were sown in pots and grow to seedling stages (with 3-4 leaves) before fresh leaf samples were taken for whole genome sequencing (Shanghai OE Biotech. Ltd, China).

The DNA was extracted using DNeasy plant mini kit (Qiagen, Hilden, Germany). The quality and concentration of DNA were tested before digested into fragments.

The library was prepared using TruSeq DNA LT Sample Prep kit (Illumina, SanDiego, CA, USA) with pair-end method.

Sequencing assembly and mapping

Fragmented DNA sample (300-500 bp) with acquired concentration was sequenced using Illumina HiSeq platform. The Illumina reads generated in this study are available at the website (http://www.ncbi.nlm.nih.gov/sra/SRP159151) with the accession number 46608_4. After separation of the raw sequence data according to the library-specific barcodes, read qualities of the sequences were initially checked using qualimap (http://qualimap.bioinfo.cipf.es/), and preprocessed by removing low-quality reads (Q20 < 70%), non-ATCG reads and short sequence reads (< 70 bp). After pre-processing, the reads were aligned against the *B. napus* reference genome v4.1 allowing default mismatches (26). A BLAST search was performed against the *Rfo* gene sequences (GeneBank Accession AJ550021) and revealed that the *Rfo* gene had high sequence identity (over 90%) with certain regions of the linkage group R_9 in *R. sativus*, leading further analysis conducted combined with both *B. napus* and R_9 from *R. sativus* . Clean reads were then aligned into self-generated reference genome which combine whole genome of *B. napus* and R09 from radish using software BWA-MEM (27). The coverage and depth of the reads aligned into R09 from radish were analyzed for the homologous regions that introgressed from R09 into *B. napus*.

Development of specific markers for CLR6430

Based on previous BLAST search results the scaffolds identified from aligned sequences in R_9 from radish in reference genome with average coverage over 115 and homologous to R_9 from radish were used for developing specific markers for CLR6430. 92 specific simple sequence repeat (SSR) primer pairs were designed based on the *Raphanus* fragment introgresssed using online marker design website: http://wsmartins.net/websat/ with default settings. The presence of the Ogu CMS and *Rfo* gene were verified in every cross generation

by PCR. Genomic DNA was extracted from young leaves of field-grown plants, using the cetyltrimethylammonium bromide (CTAB) methods (28).

Each PCR was performed in 96-well PCR plates, with each reaction containing 10-20ng/μl of genomic DNA, 1X standard PCR buffer, 2.5mM $MgCl_2$, 0.1mM dNTP, 1.0 unit Taq polymerase, 0.125μM of forward and reverse primers. PCR amplification used the following cycling parameters: 1 cycle of 5 min at 95℃; 10 cycles of 30s at 94℃,45s at 60℃ (next cycle is reduced by 0.5℃), 30s at 72℃, then followed by 30 cycles of 30s at 94℃, 45s at 55℃, 30s at 72℃ and ending with 10 min at 72℃). All PCR products were analyzed by electrophoresis in 3.0% agarose gel in 1×TAE buffer and were visualized by ethidium bromide on a digital gel documentation system.

Analysis of markers in the radish introgression

The specific markers were used to compare CLR6430 with the known rapeseed restorer R2000 (7). Parts of PCR amplification products were sequenced by Sanger sequencing, and the consensus sequences amplified from the CLR6430 and R2000 were aligned using DNAman ver3.0 (Lynnon BioSoft, Quebec, Canada).

Results

Fertility segregation identification

Ogu CMS A-lines 20QA and SC_3QA were crossed with the restorer line CLR6430 (20QA×CLR6430 and SC_3QA×CLR6430) and then selfed for F_2 and F_3. Fertility and sterility ratio of tested plants in both F2 and F3 population were closely to 1:2 (Table 1), segregated from the expected 3:1. Similarly, reciprocal crosses were conducted between CLR6430 and 20B and SC_3, and fertility and sterility ratio in the backcross population also segregated from

expectation, matched closely to 1:4 (Table 2) which distorted from the expected 1:1 ratio. Distorted segregation happened in each population possibly due to the introgressed fragment from radish, causing abnormal segregation of translocated chromosome of *B. napus* during meiosis. Meanwhile, there was no significant difference between the populations from reciprocal crosses [A(*rfrf*) × A(*Rfrf*)] and [A(*Rfrf*) × B(*rfrf*)], indicating no difference of the restorer gene (*Rfo*) passing through male or female donor. However, *Rfo*-specific markers matched precisely to their fertility status in both segregating population (F_2 and F_3) and back-crossing population (BC_1-n), indicating the fertility results from segregating populations were reliable.

Table 1 Fertility and sterility results of the offspring crossing with CLR6430 in selfing segregating populations.

Population	Generation	F_1 Female	Total	Fertility	Sterility	χ^2(P) for 1:2
A（Rfrf） selfing	F2	20QA-1	102	38	64	0.71
		SC_3QA-1	139	49	90	0.23
	F3	20QA-1	172	61	111	0.35
		20QA-2	202	72	130	0.49
		SC_3QA-1	158	51	107	0.08
		SC_3QA-2	175	53	122	0.73

Table 2. Fertility and sterility results of the offspring crossing with CLR6430 in backcrossing segregating populations.

	Crossing	Recurrent parent	Total	Fertility	Sterility	χ^2 (P) for 1:4
A(rfrf) test crossing	A(rfrf)×A（Rfrf）	20QA-1	158	31	127	0.01
		20QA-2	207	41	166	0
		SC_3QA-1	179	36	143	0
		SC_3QA-2	120	25	95	0.05
	A（Rfrf）×B(rfrf)	20B-1	158	28	130	0.51
		20B-2	243	52	191	0.3
		SC_3-1	189	37	152	0.02
		SC_3-2	195	42	153	0.29

Chromosome number and radish genome introgression

Chromosome numbers were determined in more than 30 plants of CLR6430. Majority of somatic cells (>82%) in roots had 2n=38, ranging around 38-40 (Figure 1). The radish introgression was detected using GISH with radish genomic probe and *B. napus* genome as block (Figure 2 a and b). Significant and stable red fluorescent signals were detected near centromere and satellite regions of several chromosomes, while no red signals were covered as a whole chromosome, showing CLR6430 was introgressed with radish genome without additional radish chromosome.

Figure 1 Chromosome determination of CLR6430 under 40X microscope. More than 82% of detected chromosome numbers were 2n=38 of 30 plants from two generation of CLR6430. Scale bar = 10 µm.

Restorer gene was detected by FISH using C genome and BAC fish probe while A genome of *B. napus* as block (Figure 2 c). Green signals were detected consistently in A genome not C genome of *B. napus*, which is different from previous restorer lines (7, 21, 23).

Figure 2 Fluorescent *in situ* hybridization (FISH) analysis of radish-derived Ogu CMS restorer of *B. napus* (CLR6430). DAPI (blue) and merged signals (red signals from *R.sativus* probe) from somatic cells are shown in (a) and (b). (a) CLR6430 using 10X *B. napus* genome as block; (b) CLR6430 using 20X *B. napus* genome as block; (c) DAPI (blue) and merged (red signals from C genome of *B. napus* and green signals from restorer gene directed by arrows) from anther of CLR6430 is shown. Scale bar = 10 µm.

Re-sequencing of CLR6430 and its genomic assembly

In total, we obtained 134.338 Gb of pair-end sequencing data with a high-quality base ratio of 98.2%, and 71.6% of the reads aligned to the reference with an average coverage of more than 50X (Table 3). While de novo assembling using SOAPdenovo2, all reads were assembled into 568,120 scaffolds and 641,453 contigs (sequence reads >500 bp were summarized).

Table 3. Basic sequencing results of CLR6430

	R1-fastq	R2-fastq
Total reads number	447,797,399	447,797,399
Clean Reads	430,273,428	430,273,428
Clean eads ratio	96.09%	96.09%
Total base number	67169609850(67.169G)	67169609850(67.169G)
Base number in clean reads	64541014200(64.541G)	64541014200(64.541G)
High quality base number in clean reads	64031420594(64.031G)	62812421023(62.812G)
High quality base ratio in clean reads	99.21%	97.32%

Specific molecular marker development

The re-sequencing mapping and alignment results revealed homologous reads derived from R9 in radish assembled into R9 of "reference genome" and the average coverage of the region between 66.13 (Rsa1.0_00994.1) and 138.53cM (Rsa1.0_00176.1) was over 115, much higher than the other regions in R9 group, indicating a high possibility of introgressed fragment from radish into CLR6430. The sequences of this region were then used for developing specific molecular markers in segregating population (Figure 3). Considering the conservation and variability of SSR markers between the two species, sequences from introgressed fragment were used to develop SSR markers for marker-assisted breeding. Thirty-two specific SSR markers were developed and verified with corresponding fertility condition in field. Detailed information of specific SSR markers (Table 4) and sequence of amplified PCR products from Table 4 were presented in Additional Table 1.

Figure 3 PCR products amplified by specific SSR markers CLR9-16 in F_2 segregating population. "S" and "F" refer to the samples identified with sterility or fertility during flowering time.

Table 4. Specific primer pairs designed from the introgressed region from radish in CLR6430.

Marker Name	Forward（5'→3'）	Reverse（5'→3'）	Size of Produc
CLR9-1	GAACTTATGGCACTCCGATCT	CGAAGCAAGTAAGAAACACAC	323
CLR9-2	GGTCATTTTCTTCCTTGATAGC	ATATAACTAGGTGTTTTGCCCG	354
CLR9-3	ATATCGCACGGGTTCCTTAC	CATCCATCAGTTCAATCGGTTA	439
CLR9-4	TGTTAAAACCGAGGGAAAAG	TCTGCACTTGGGTCACTACAAT	364
CLR9-5	CGTAGGGCAGCTTTGATTTTA	GCTTGTACGCTTCTTCCAGATT	367
CLR9-6	AAAGAAGTCTCGCCTGAACA	ATGAGAATGGCTAGTCCGGTTA	349
CLR9-7	AAGAGAAAACCAGAGCGACA	GCAGCGATAGGAAATTGGATAA	364
CLR9-8	GCAGCGATAGGAAATTGGATA	AAGAGAAAACCAGAGCGACA	374
CLR9-9	CGAACAGAATTGAAACCGAA	GTTGTACGTCTTCCACTTTCCC	392
CLR9-10	CAGAAGCAAGTCGAGAGAGA	AGGAACCGACATTCAGAGAGA	348
CLR9-11	TATAAAACCTGGGGATTGTTG	AATTAACCTTGTCGGGTGAAGA	369
CLR9-12	AAATGCCTTCCTTGATAACTG	CGAAGATTTCATTGCTGATACG	360
CLR9-13	TGGTGGTGTCTCAAAATGGTA	CATGGTACTCCTGAGCTTATTT	385
CLR9-14	TGCTTTGTATTCATCTCTCCCC	CGACTCTTCAATGTGCATCTCT	367
CLR9-15	TGCTAGGGTTCCTCTGGATCT	TCTCCTTCAAAGCAATCTCTCC	366
CLR9-16	CTGAGAGGATCATGTTTTGTG	GCAGAGACTTCTTCACCGTCTT	311
CLR9-17	CTTTATCTGCTTCTGCTGTTGC	TTTCTCCCTGATGACCTTTTGT	346
CLR9-18	AATAGCTTCCTCACCTGTCAC	GGTTTAGACGGCACCTAGTCAG	346
CLR9-19	TGCATACAAACCGAGAATCA	CGGTCTAACATATTGCACATTC	343
CLR9-20	GGACAAACAAGGATGGAGTT	CCAAATCTGAATGCGAGAGAAT	535

Comparison of the introgression between CLR6430 and R2000 with markers

The above-mentioned 32 markers were used to characterize the CLR6430 in comparison with R2000. The minimum size of the introgressed fragment in CLR6430 was estimated at 72.14 CM (between Rsa1.0_00994.1 and Rsa1.0_00071.1). Comparison to CLR6430, R2000

had five continuously lost markers (CLR9-20, CLR9-21, CLR9-26, CLR9-27, CLR9-30,) (Table 5), estimating the introgressed region about 48.48 CM (between Rsa1.0_00994.1 and Rsa1.0_00045.1). And the size of fragments amplified by primers pairs CLR9-1, CLR9-6, CLR9-7, CLR9-10 and CLR9-31from CLR6430 and R2000 were different. Besides, the amplified PCR sequences by the new developed SSR markers in this study revealed significant differences between CLR6430 and R2000, presenting various sequence insertion, deletion and replacements (Figure 4).

Table 5. Specific marker comparison for CLR6430, R2000 and radish in this study. "+" and "-" were used to describe the results as positive or negative. "Baiyuchun" was the name of a radish cultivar.

Radish genome	Region (cM)	Marker Name	Scaffold Number	CLR6430	R2000	Baiyuchun	Note
R9	66.13	CLR9-32		-	-	+	
R9	66.97	CLR9-31		+	+	+	
R9	72.64	CLR9-13		+	+	+	
R9	74.73	CLR9-11	Scaffold510	+	+	+	
R9	76.06	CLR9-33		+	+	+	
R9	76.06	CLR9-34		+	+	+	
R9	84.38	CLR9-10		+	+	+	
R9	93.38	CLR9-14	Scaffold4378	+	+	+	*Rfo* region
R9	93.38	CLR9-1	Scaffold662<br>Scaffold33209<br>Scaffold42248<br>Scaffold61106<br>Scaffold176555<br>Scaffold251659	+	+	+	*Rfo* region
R9	93.38	CLR9-2		+	+	+	*Rfo* region
R9	93.38	CLR9-3	Scaffold80976	+	+	+	*Rfo* region
R9	93.38	CLR9-4	Scaffold66484	+	+	+	*Rfo* region
R9	93.38	CLR9-5	Scaffold6789	+	+	+	*Rfo*

			Scaffold208888				region
R9	93.38	CLR9-22		+	+	+	*Rfo* region
R9	93.38	CLR9-23	Scaffold80	+	+	+	*Rfo* region
R9	93.38	CLR9-6	Scaffold80 Scaffold100822	+	+	+	*Rfo* region
R9	93.38	CLR9-7	Scaffold80 Scaffold214757	+	+	+	*Rfo* region
R9	93.38	CLR9-8	Scaffold80 Scaffold214757	+	+	+	*Rfo* region
R9	93.38	CLR9-9	Scaffold80	+	+	+	*Rfo* region
R9	94.92	CLR9-24		+	+	+	
R9	94.92	CLR9-25		+	+	+	
R9	103.77	CLR9-15	Scaffold10090 Scaffold111532	+	+	+	
R9	115.45	CLR9-16	Scaffold7186 Scaffold53692	+	+	+	
R9	115.45	CLR9-17	Scaffold62	+	+	+	
R9	115.45	CLR9-18	Scaffold4699	+	+	+	
R9	115.45	CLR9-19	Scaffold62	+	-	+	
R9	118.34	CLR9-20		+	-	+	
R9	119.83	CLR9-21		+	-	+	
R9	120.43	CLR9-26	Scaffold13452	+	-	+	
R9	121.78	CLR9-27		+	-	+	
R9	130.79	CLR9-28		+	+	+	
R9	132.17	CLR9-29	Scaffold83707	+	+	+	
R9	138.23	CLR9-30		+	-	+	

Figure 4 Sequence comparison between CLR6430 and R2000 in the region near *Rfo* gene, which was amplified by CLR9-7 markers.

Discussion

The radish-derived male sterility has the advantage of complete and stable sterility in *B. napus* and is favored by breeders in commercial production (29). However, creation of an Ogu-CMS restorer line in *B. napus* remains difficult as no restorer genes were directly found

in this species, nor in some Asian radish lines. Previous research outlined the difficulties and effort in the way of producing an Ogu-CMS *B. napus* restorer line (11), either through cell fusion or consistent backcrossing. In a previous study, Chen *et al* (24) used a *Raphnobrassica* (AACCRR) as donor and successfully obtained the hybrid with *B. napus* through grafting to overcome the incompatibility between these two parents. After 15 years of intensive selection, a homozygous (*RfRf*) restorer line CLR650 was finally obtained. Similar to previous early-version Ogu-CMS restorer line, CLR650 was associated with deleterious agronomic traits, such as high level of glucosinolate and low seed production. Updated version CLR6430 was generated through multiple generations of back-crossing and selfing, and further corresponding analysis of fertility models, chromosome behaviors, and genomic re-sequencing-based specific markers development were characterized in this study to provide basic information for utilizing of Ogu-CMS restorer line.

Generally, introgressed fragment from radish is the key part for understanding of the newly created Ogu-CMS in *B. napus*. In previous published lines, at least 50 cM of radish genome was integrated into restored rapeseed based on AFLP marker information (30). They identified the region of Arabidopsis genome syntenic to the *Rfo* locus, then carried out fine mapping of the *Rfo* gene in a segregating radish population to find the likely ortholog of the *Rfo* gene. In recent years, along with the development of second-generation sequencing, radish genome has also been available (31-33) and useful for the development of specific markers for breeding purposes. In our study, CLR6430 was re-sequenced and aligned against a combined reference genome, which was the whole *B. napus* genome plus an additional chromosome from radish that contained the *Rfo* gene. The high coverage sequences

assembled into R9 in the reference genome indicating the most likely homologous regions from radish introgressd into *B. napus*, which is the region between 66.13 (Rsa1.0_00994.1) and 138.53cM (Rsa1.0_00176.1) Therefore, there is about 72.4 cM of radish genome integrated into CLR6430. And the following design of SSR markers for CLR6430 was also based on the re-sequencing and alignment results, providing solid and more precise information for later improvement and utilization of CLR6430.

Secondly, creation of an ideal restorer line for Ogu-CMS *B. napus* remains difficult. BAC-FISH results (Figure 2) has demonstrated restorer gene from Radish located in A genome rather than N19 from C genome as previously reported (7, 21, 23), therefore, the introgression of radish genome into *Brassica napus* is different. After obtaining homozygous (*RfRf*) instead of heterozygous (*Rfrf*) in the loci of the restorer gene, we still have to break the linkages with some deleterious traits such as high glucosinolate content and self-incompatibility. R2000, the widely accepted Ogu-CMS restorer line developed by INRA, has been utilized for three-line system production of hybrid rapeseed.

Meanwhile, the comparison between CLR6430 and R2000 in this study has shown significant differences, revealing the different origin. CLR6430 has improved performances in terms of glucosinolate content and selfing ability compared to its earlier version CLR650, while the introgressed fragment from radish remain long and require further improvement. In this case, developed markers for R2000 or other reported Ogu-CMS restorer were not applicable for CLR6430, hence self-designed markers based on resequencing results are meaningful for further selection and improvement for CLR6430.

Besides, segregation distortion was found in CLR6430 in selfing and backcrossing

populations. Distorted segregation is the deviation of the observed genotypic ratios from the expected frequencies based on Mendelian's laws of inheritance, which is considered as an evolutionary force and associated with genetic factors involved in reproduction and fitness (34). Several mechanisms of segregation distortion have been reported in plants, such as chromosomal rearrangement and genomic interactions causing zygotic abortion, hybrid sterility, haploid induction and restriction of gene introgression (35). In our case, distorted segregation happened stably in each population possibly due to the introgressed fragment from radish, causing abnormal segregation of translocated chromosome of *B. napus* during meiosis, which could affect the estimation of map distances and the order of markers when many distorted markers are presented. Other reason for this phenomenon could be the selection of both CLR650 and CLR6430 were based on high-pressure of *Rfo* gene and the introgressed fragment along it remains large.

The radish-derived male sterility has the advantage of complete and stable sterility in *B. napus* and was favored by breeders in commercial production (36). As one of the most important rapeseed market, China has rarely utilized this Ogu-CMS system in hybrid seed production, mainly due to the lack of self-developed restorer line and patent restrictions applied from other restorer sources such as R2000 and SRF developed by INRA and Pioneer Hibred, respectively. Making good use of CLR6430 could be the breakthrough for future application of Ogu-CMS system in this market.

In summary, the DNA sequence surrounding the *Rfo* in CLR6430 is different from that of R2000, and resequencing-based *Rfo*-related markers presented a more efficient method for the improvement of CLR6430. However, the glucosinolate content remains higher than market

level, and marker-assisted improvement still at early stage for seed settings and other agronomic traits. Moreover, the genetic mechanisms underlying the observed segregation distortion for *Rfo* in the inbreeding and backcross offspring requires further investigation. Further studies are required to locate and reduce the size of the introgression in CLR6430 for better breeding outcomes.

References

1. Fu T. Some investigation on polima cytoplasmic male sterility in *Brassica napus*. Eucatpia Cruciferae Newsletter. 1987;12(1):46-7.
2. Ogura H. Studies on the new male-sterility in Japanese radish,with special reference to the utilization of this sterility towardsthe practical raising of hybrid seeds. Memoirs of the Faculty of Agriculture, Kagoshima University. 1968;6:39-78.
3. Shiga T, Baba S. Cytoplasmic male sterility in rape plants (*Brassica napus* L.). Japanese Journal of Breeding. 1971;21:16-7.
4. Kang L, Li P, Wang A, Ge X, Li Z. A novel cytoplasmic male sterility in *Brassica napus* (inap CMS) with carpelloid stamens via protoplast fusion with Chinese woad. Frontiers in Plant Science. 2017;8:529.
5. Sovero M. Cytoplasmic male sterility in turnip-rape (*Brassica campestris* L.): University of Manitoba; 1987.
6. Heyn F. Transfer of restorer genes from Raphanus to cytoplasmic male sterile *Brassica napus*. Cruciferae Newsletter. 1976;1:15-6.
7. Primard-Brisset C, Poupard JP, Horvais R, Eber F, Pelletier G, Renard M, et al. A new recombined double low restorer line for the Ogu-INRA cms in rapeseed (*Brassica napus* L.). Theoretical and Applied Genetics. 2005;111(4):736-46.
8. Sakai T, Liu HJ, Iwabuchi M, Kohno-Murase J, Imamura J. Introduction of a gene from fertility restored radish (*Raphanus sativus*) into *Brassica napus* by fusion of X-irradiated protoplasts from a radish restorer line and iodacetoamide-treated protoplasts from a cytoplasmic male-sterile cybrid of *B. napus.* . Theor Appl Genet. 1996;93(3):373-9.
9. Pelletier G, Primard C, Vedel F, Chetrit P, Renard M, Pellandelourme R, et al., editors. Molecular, phenotypic and genetic characterization of mitochondrial recombinants in rapeseed. 7th International Rapeseed Congress; 1988; Poznan, Pologne.
10. Delourme R, Bouchereau A, Hubert N, Renard M, Landry BS. Identification of RAPD markers linked to fertility restorer gene for the Ogura radish cytoplasmic male sterility of rapeseed (*Brassica napus* L.). Theor Appl Genet. 1994;88(6):741-8.
11. Delourme R, editor Double low restored F1 hybrids can be produced with the Ogu-INRA CMS in rapeseed. The 10th International Rapeseed Congress; 1999; Canberra, .
12. Charne DG, Grant I, Kraling K, Patel JD, Pruvot JCM, Tulsieram LK, inventorsOilseed *Brassica*

containing an improved fertility restorer gene for ogura cytoplasmic male sterility. United States2002.

13. Pleines SC, Stiewe RK, Brummermann K, Gielen JJL, inventorsFertility restoration for Ogura cytoplasmic male sterile *Brassica* and method. United States2007.

14. Zhang Y, Patel J, Tulsieram L, inventors; US, assignee. *Brassica* ogura restorer lines with shortened raphanus fragment (SRF)2009.

15. Brown GG, Nata F, Hua J, Wargachuk R, Dendy C, Patil P, et al. The radish *Rfo* restorer gene of Ogura cytoplasmic male sterility encodes a protein with multiple pentatricopeptide repeats. Plant Journal. 2003;35(2):262-72.

16. Desloire S, Gherbi H, Laloui W, Marhadour S, Clouet V, Cattolico L, et al. Identification of the fertility restoration locus, Rfo, in radish, as a member of the pentatricopeptide-repeat protein family. EMBO reports. 2003;4(6):588-94.

17. Feng J, Primomo V, Li Z, Zhang Y, Jan CC, Tulsieram L, et al. Physical localization and genetic mapping of the fertility restoration gene Rfo in canola (*Brassica napus* L.). Genome. 2009;52(4):401-7.

18. Wang ZW, Zhang YJ, Xiang CP, Mei SY, Zhou Y, Chen GP, et al. A new fertility restorer locus linked closely to the Rfo locus for cytoplasmic male sterility in radish. Theoretical and Applied Genetics. 2008;117(3):313-20.

19. Wang ZW, Zhang LJ, Chen J, Xiang CP, Mei SY, Zhou Y, et al. A chimeric *Rfo* gene generated by intergenic recombination cosegregates with the fertility restorer phenotype for cytoplasmic male sterility in radish. Molecular Breeding. 2010;25(2):339-49.

20. Wang ZW, Wang C, Gao L, Mei SY, Zhou Y, Xiang CP, et al. Heterozygous alleles restore male fertility to cytoplasmic male-sterile radish (*Raphanus sativus* L.): a case of overdominance. Journal of Experimental Botany. 2013;64(7):2041.

21. Hu X, Sullivan-Gilbert M, Kubik T, Danielson J, Hnatiuk N, Marchione W, et al. Mapping of the Ogura fertility restorer gene Rfo and development of Rfo allele-specific markers in canola (*Brassica napus* L.). Molecular Breeding. 2008;22(4):663-74.

22. Tian E, Roslinsky V, Cheng B. Molecular marker-assisted breeding for improved Ogura cms restorer line (*RfoRfo*) and mapping of the restorer gene (*Rfo*) in *Brassica juncea*. Molecular Breeding. 2014;34(3):1361-71.

23. Yu HL, Fang ZY, Liu YM, Yang LM, Zhuang M, Lv HH, et al. Development of a novel allele-specific Rfo marker and creation of Ogura CMS fertility-restored interspecific hybrids in *Brassica oleracea*. Theor Appl Genet. 2016;42(3):1-13.

24. Chen W, Li M, Wang T, Hui R, Tu J. Development of new restorer materials with Ogu CMS in *Brassica napus*. Scientia Agricultura Sinica. 2012;45(8):1465-74.

25. Ge X, Li Z. Intra- and intergenomic homology of B-genome chromosomes in trigenomic combinations of the cultivated *Brassica* species revealed by GISH analysis. Chromosome Research. 2007;15(849-861).

26. Chalhoub B, Denoeud F, Liu S, Parkin I, Tang H, Wang X, et al. Plant genetics. early allopolyploid evolution in the post-Neolithic *Brassica napus* oilseed genome. Science. 2014;345(6199):950.

27. Li H, Durbin R. Fast and accurate short read alignment with Burrows-Wheeler transform. Bioinformatics. 2009;25:1754 - 60.

28. Saghai-Maroof MA, Soliman KM, Jorgensen RA, Allard RW. Ribosomal DNA spacer-length polymorphisms in barley: mendelian inheritance, chromosomal location, and population dynamics. Proceedings of the National Academy of Sciences of the USA. 1984;81:8014-8.

29. Havey MJ. The use of cytoplasmic male sterility for hybrid seed production. In: Daniell H, Chase C,

editors. Molecular biology and biotechnology of plant organelles. Netherlands: Springer 2004. p. 623-34.
30. Giancola S, Marhadour S, Desloire S, Clouet V, Falentin-Guyomarc'H H, Laloui W, et al. Characterization of a radish introgression carrying the Ogura fertility restorer gene Rfo in rapeseed, using the Arabidopsis genome sequence and radish genetic mapping. Theoretical and Applied Genetics. 2003;107(8):1442-51.
31. Kitashiba H, Li F, Hirakawa H, Kawanabe T, Zou Z, Hasegawa Y, et al. Draft sequences of the radish (*Raphanus sativus* L.) genome. DNA Research. 2014;21(5):481-90.
32. Mitsui Y, Shimomura M, Komatsu K, Namiki N, Shibata-Hatta M, Imai M, et al. The radish genome and comprehensive gene expression profile of tuberous root formation and development. Scientific Reports. 2015;5:10835.
33. Zhang X, Yue Z, Mei S, Qiu Y, Yang X, Chen X, et al. A de novo genome of a Chinese radish cultivar. Horticultural Plant Journal. 2015;1(3):155-64.
34. Taylor DR, Ingvarsson PK. Common features of segregation distortion in plants and animals. Genetica. 2003;117(1):27-35.
35. Manriquecarpintero NC, Coombs JJ, Veilleux RE, Buell CR, Douches DS. Comparative analysis of regions with distorted segregation in three diploid populations of potato. G3 Genesgenetics. 2016;6(8):2617-28.
36. Havey M. The use of cytoplasmic male sterility for hybrid-seed production. Molecular Biology & Biotechnology of Plant Organelles. 2004:623-34.

Acknowledgements

We thank Prof Tingdong Fu for his generosity of providing the initial bridging material and his valuable advice on the progress. We also thank Ms. Qian Yang for her assistance in data analysis of the resequencing data of CLR6430.

Author Contributions Statement

WTH and GYM conducted the study, analyzed the data and prepared the manuscript. CWJ and LM bred the initial Ogu-CMS restorer line CLR650. WZX and XSQ performed on GISH and FISH experiments. HSJ provided and contributed on the comparison with previous Ogu-CMS restorer line R2000. TJX reviewed and edited the manuscript. All authors read and approved the manuscript.

Competing Interests

The authors declare no competing interests.

Funding Statement

Funding for operating costs of this research was provided both by the National Natural Science Foundation for Young Scientists of China (Grant No. 31401467) and The Key Research and Development Program of Hunan Province (Grant No. 2016NK2200).

Data Accessibility

The data discussed in this publication have been deposited to NCBI's SRA database with BioProject number PRJNA488570 and is accessible through the website linkage at: http://www.ncbi.nlm.nih.gov/sra/SRP159151.

Figure Legends

Figure 1 Chromosome determination of CLR6430 under 40X microscope. More than 82% of detected chromosome numbers were 2n=38 of 30 plants from two generation of CLR6430. Scale bar = 10 μm.

Figure 2 Fluorescent *in situ* hybridization (FISH) analysis of radish-derived Ogu CMS restorer of *B. napus* (CLR6430). DAPI (blue) and merged signals (red signals from *R.sativus* probe) from somatic cells are shown in (a) and (b). (a) CLR6430 using 10X *B. napus* genome as block; (b) CLR6430 using 20X *B. napus* genome as block; (c) DAPI (blue) and merged (red signals from C genome of *B. napus* and green signals from restorer gene directed by

arrows) from anther of CLR6430 is shown. Scale bar = 10 μm.

Figure 3 PCR products amplified by specific SSR markers CLR9-16 in F_2 segregating population.

Figure 4 Sequence comparison between CLR6430 and R2000 in the region near *Rfo* gene, which was amplified by CLR9-7 markers

Supplementary Information

Supplementary Table 1: Sequence of amplified PCR products from self-designed SSR markers from Table 4.

Figure 1

Click here to download Figure Figure 1.png

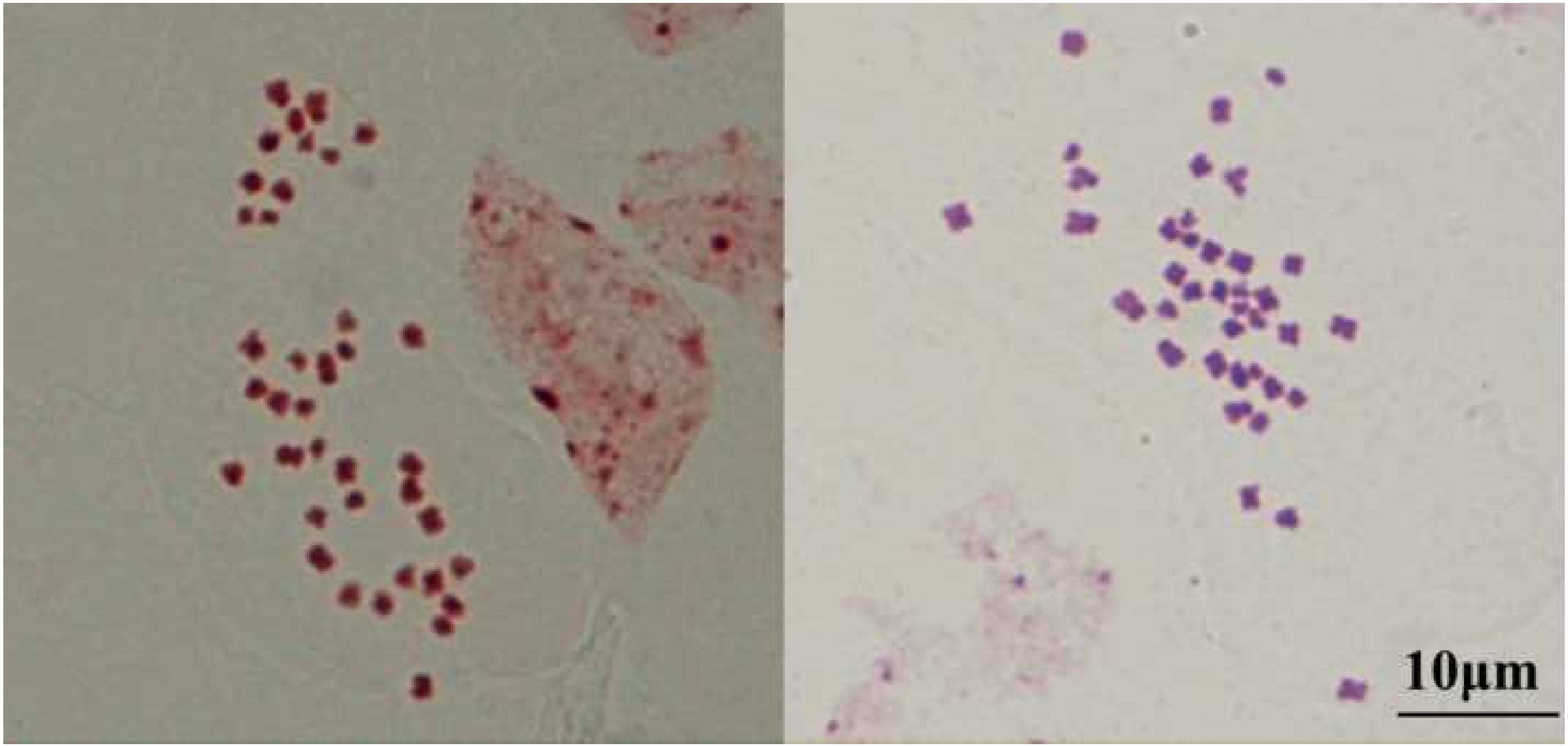

Click here to download Figure Figure 2.png

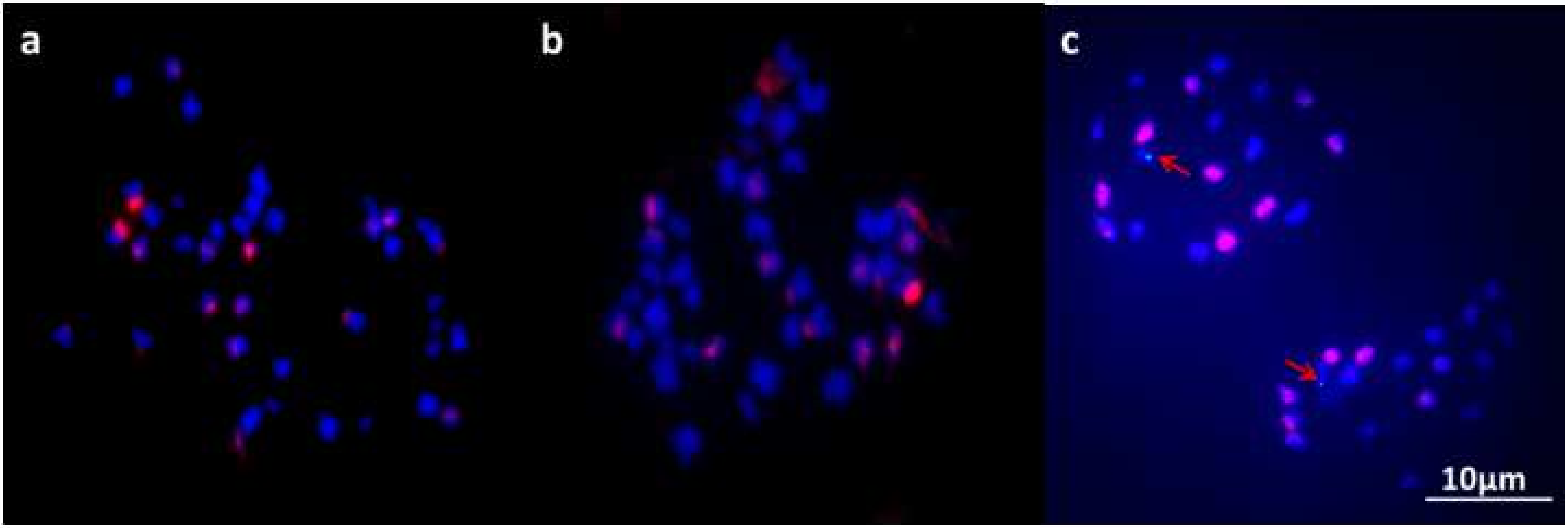

Figure 3

Click here to download Figure Figure 3.jpg

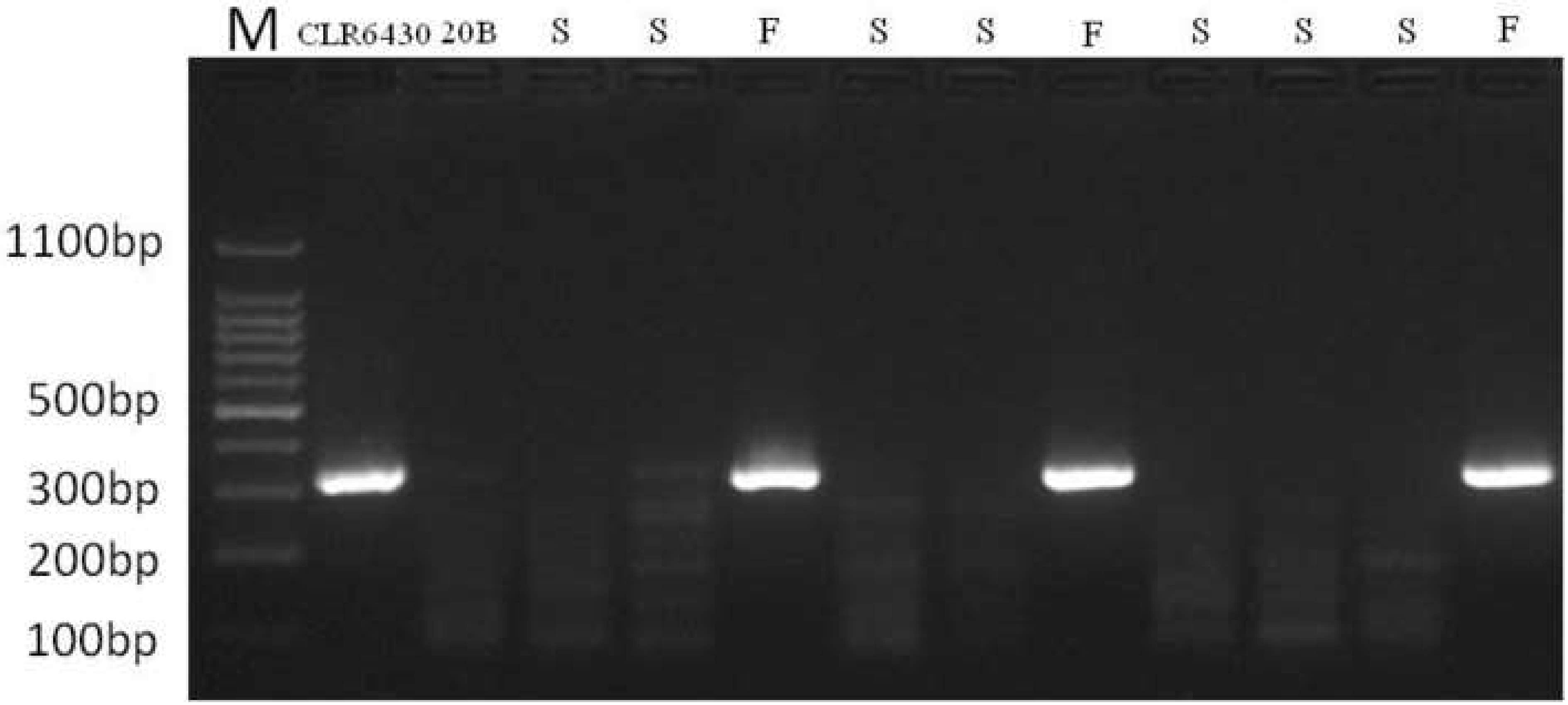

Figure 4 Click here to download Figure Figure 4.png

```
CLR6430   ....CCGAAGGGAACAAAAGAAGGAAAACGAAAAATAAAA    36
R2000     CCGACGGGGGGAAAAAAGAACCATAAAATGCAACCCAAAT    40

CLR6430   AAAAG..AAGTTAAGACATCTTTTTCTTTTGTTAATATAA    74
R2000     TCTTTTTGATTCGAGGCCATCACTTTTTTTTTTATTATTA    80

CLR6430   ATAAACAACCAGACCAACATTGATCTGCCTTTTCCTTTGT   114
R2000     AAACCCAACCCCCCAAAATTGCCCTTGCATTTTTCTTCTT   120

CLR6430   GGATTAAAATCAATTGGGCCACCAAAATCAAATAATCCCC   154
R2000     GGGATTC..TCAATTTGTCCCCCCT..CCAAAGAATCCCC   156

CLR6430   AATCATGGGTATTAATCCCTTCTGGAGGCCCCACTCAATT   194
R2000     AAAAATCGGGTTTAAAAAATGACGGAGCCCCCCCTCCAAA   196

CLR6430   TTGCCCAACAAAAAAATCGCTTT.ACTTTTTTTGCTTTGT   233
R2000     AATCACAAAAAAAAAAAGGCTTTGACCCTTTTTGCGGTTG   236

CLR6430   TTATTTTTTTATATTACCAAAAAAAAAAAATTTTTTCCCC   273
R2000     ATAGATTTAAAAAAAAA.AAAAAAAAAAAATTTTTTCCCC   275

CLR6430   ATTTTAATTAAAAGGGAAAAGCGGGAAAATAAAAATTTCT   313
R2000     AATTTAA..AAAAGGATTAAAAGGAAAACCAAAAATTTTT   313

CLR6430   TTTTTTTTTATTA...TGGGGTCTAACCCTTTTCCTTTGG   350
R2000     TCCTTTTTTTTTTGGTTGGGTTTTACCCCTTTTCCTTTGC   353

CLR6430   TTTAAATTAAAAAA..........................   364
R2000     TTCAAATTAAAAAAAGCTTTCAAGCGGCTGCAAAACACTT   393

CLR6430   ........................................   364
R2000     GATGAGTCCATCACAAGCGTCTTAGAAAGGGTGACACAAA   433

CLR6430   .........                                  364
R2000     GGGTGTATG                                  442
```

Additional Table 1

Click here to access/download
Supporting Information
Additional Table 1.docx
