# Supplementary material for: Genetic characterization of a new radish introgression line carrying the restorer gene for Ogura CMS in Brassica napus
Source: PLoS One. 2020 Jul 28;15(7):e0236273. doi: 10.1371/journal.pone.0236273 (PMC7386589; doi:10.1371/journal.pone.0236273)
Supplement: Attachment — Submitted filename: Plos_one Response to the reviewers.docx [file pone.0236273.s003.docx]

Dear Editor,

Thank you for the opportunity for us to revise and resubmit our manuscript to PLOS ONE. We have responded to each of the comment by the reviewers which we found very helpful. We also have gone through the manuscript thoroughly again to minimize the errors in grammar and spelling. The responses to each reviewer are detailed below.

Please contact us if there are more queries.

Kind regards,

Tonghua Wang and Yiming Guo (for the authors)

Reviewer #1: In their study, Guo et al. present an investigation into the Rfo introgression in CLR6430. Unlike other restorer line, they find Rfo on N19 (C9?) in a differently sized introgression.
I can't find any fault with the general outline of the study or the findings, only minor things (below).

It seems that the short reads generated in this study are openly available; however, the genome assembly generated is not. Will the authors deposit this assembly somewhere?
Thank you for these positive comments and careful reminder. We have deposited the genome assembly to the NCBI database under the same BioProject number PRJNA488570.

Some minor things:
Line 29, typo, 'introgresssed'

Revised to “introgressed”
Line 170 - the link in the PDF is broken and points to '<http://wsmartins.net/websat/> with default', should be without the 'with default'

The website has been updated and now changed to ‘http://200.137.197.254/~wellington/websat/’,
Line 218 - incomplete sentence? 'Restorer gene was detected by FISH using C genome and BAC fish probe while A genome of B. napus as block'

Revised to ‘Restorer gene was detected by FISH using C genome and BAC clone as probes while A genome of *B. napus* as block’
Line 233, Table 3 - typo, 'Cleaned eads'

Revised to ‘clean reads’
Line 265, why is Baiyuchun mentioned only once here? Why is it not in the methods?

The relative information has added into the methods part.
Line 287, typo - 'Raphnobrassica'

Revised to ‘Raphanobrassica’.
There are a few more smaller typos which should have been caught by rigorous spell checking.

We have gone through the manuscript thoroughly to minimize the errors in grammar and spelling.

Reviewer #2: This manuscript 'Genetic characterization of a new radish introgression line carrying the restorer gene
for Ogura CMS in Brassica napus' is based on well defined problem and conducted in a profound way. It is a significant improvement from the earlier available studies. This should be accepted for publication after incorporating the following corrections:

i. Introduction: Explain the importance of restores in oilseed brassicas
One sentence explaining the importance of restores in oilseed *Brassicas* has been added into the Introduction part.
ii. In the materials and method, the population developed using the CMS lines and restores and maintainers with restorers were mentioned. However, it has not been made clear how these population were used for this study. The method used in Table 5 is not mentioned clearly in this section.
Thank you for the reminder, the information of the radish cultivar “Baiyunchun” has been added in the methods part for Table 5.
iii. Results: Explained appropriately, however, it is difficult to understand how the different populations were used in validation of the designed markers and segregation ratio. Explain these portion elaborately.
The process of validation of designed markers in tested and verified in the F2 segregating population was further explained in the context.
iv. Discussion: This section need improvement in terms of the works done and its significance. This part should explain all the experiments conducted supported by sufficient number of citations.
Thank you for the suggestion. We revised and rearranged several parts in this part while added a few more citations.
Comments in the manuscript is also attached for further refernce.
